# Survival rate and Outcome of extracorporeal life support (ECLS) for treatment of acute cardiorespiratory failure in trauma patients

**DOI:** 10.1038/s41598-019-49346-z

**Published:** 2019-09-09

**Authors:** Nikolaus W. Lang, Ines Schwihla, Valerie Weihs, Maximilian Kasparek, Julian Joestl, Stefan Hajdu, Kambiz Sarahrudi

**Affiliations:** Department of Orthopaedics & Trauma Surgery, Medical University of Vienna, Waehringer Guertel 18–20, A-1090 Vienna, Austria

**Keywords:** Outcomes research, Risk factors

## Abstract

Extracorporeal life support (ECLS) remains the last option for cardiorespiratory stabilization of severe traumatic injured patients. Currently limited data are available and therefore, the current study assessed the survival rate and outcome of ECLS in a Level I trauma center. Between 2002 and 2016, 18 patients (7 females, 11 males) with an median age of 29.5 IQR 23.5 (range 1–64) years were treated with ECLS due to acute traumatic cardiorespiratory failure. Trauma mechanism, survival rate, ISS, SOFA, GCS, GOS, CPC, time to ECLS, hospital- and ICU stay, surgical interventions, complications and infections were retrospectively assessed. Veno-arterial ECLS was applied in 15 cases (83.3%) and veno-venous ECLS in 3 cases (16.6%). Survivors were significant younger than non-survivors (p = 0.0289) and had a lower ISS (23.5 (IQR 22.75) vs 38.5 (IQR 16.5), p = n.s.). The median time to ECLS cannulation was 2 (IQR 0,25) hours in survivors 2 (IQR 4) in non-survivors. Average GCS was 3 (IQR 9.25) at admission. Six patients (33.3%) survived and had a satisfying neurological outcome with a mean GOS of 5 (IQR 0.25) (p = n.s.). ECLS is a valuable treatment in severe injured patients with traumatic cardiorespiratory failure and improves survival with good neurological outcome. Younger patients and patients with a lower ISS are associated with a higher survival rate. Consideration of earlier cannulation in traumatic cardiorespiratory failure might be beneficial to improve survival.

## Introduction

Severe trauma in combination with thoracic injuries are accompanied by life-threatening conditions including cardiorespiratory failure^1^. The main reasons for traumatic cardiac arrest due to blunt (96,1%) or penetrating (3,9%) thoracic trauma are: hypovolaemia (48%), hypoxaemia (13%), tension pneumothorax (13%) and cardiac tamponade (10%)^2,3^. Advanced life support (ALS) due to acute thoracotomy with cross-clamping of the aorta and open cardiac massage had shown poor results in the past^4–6^. Severe trauma can also cause acute respiratory distress syndrome (ARDS) in up to 10% of the patients^7^. The common causes for traumatic ARDS mostly occurs in blunt thoracic trauma accompanied by pulmonary contusion, hypovolaemic shock, massive transfusion, flail chest due to reanimation, and an ISS (Injury Severity Score) >25^8,9^. Extracorporeal life support (ECLS) remains the last option for acute cardiorespiratory stabilization in these patients^5,7,10–12^. However, there are only limited data on the veno-arterial or veno-venous ECLS as emergency treatment in traumatic cardiac arrest or hypoxemia^5,12–16^.

Therefore, the current study aims to report our experience on the ECLS treatment in trauma patients and addresses the following questions: (1) Which extent of injury severity makes the use of ECLS necessary. (2) How long is in average the time between trauma and ECLS-cannulation? (3) How many ECLS related complications occurred? (4) How is the neurological outcome in trauma patients requiring ECLS treatment?

## Results

### Clinical presentation

Eight patients were admitted to the trauma room awake or minimally sedated, respiratory stable under application of 2–4 liters of using an oxygen mask and i.v. fluid supply. Nine patients were admitted intubated, mechanically ventilated and hemodynamically stable. Out of these patients 3 became hypoxemic accompanied with rapidly decreasing peripheral oxygen saturation of <85% as well a PaCO_2_ > 115 mmHg/PaO_2_ < 50 mmHg in the blood gas analysis (BGA). Fourteen patients became hemodynamically instable and required advanced life support. At least one patient arrived in the trauma room intubated and under on going mechanical resuscitation and advanced life support.

More information on clinical presentation in Table 2.

### Injury severity & survivors

One third of all patients (n = 6) that required ECLS due to acute cardiorespiratory failure survived. For further analysis, patients were allocated to survivors and non-survivors. The mean follow up was 4.8 ± 2.3 years (range 52–407 weeks). Cannulation for ECLS lead to ROSC (return of spontaneous circulation) in ten patients (55.6%) and was not successful in eight patients (44.4%). However, four patients (22.2%) died within 36 hours- 45 days on Intensity care unit (ICU) due to multi organ failure (MOF) or ICU related complications such as sepsis.

The median ISS at admission was 34.5 (IQR 26.5, range 16–50). Survivors were significantly younger than non-survivors (p = 0.0289) and had a lower ISS (23.5 IQR 22.75 vs 38.5 IQR 16.5, p = n.s.) The median SOFA score was 12 (IQR 4, range 5–15) in all patients and was comparable between survivors and non-survivors (p = n.s.).

### ECLS procedure related results

Cannulation for ECLS was performed within 2 (IQR 3, range 0.5–6) hours in median from initial trauma. The median time to ECLS cannulation in survivors was 2 (IQR 0.25) hours compared to 2 (IQR 4) hours in non-survivors (p = n.s.). Earlier cannulation for ECLS (<3 hours) had no influence on overall survival (p = n.s.). The median duration of ECLS was in non-survivors 0.5 (IQR 27, range 0–264) hours and 48 (IQR 54, range 24–96) hours in survivors. The mean ICU stay was 32.7 days (±11.3) days and the average hospital stay until discharge was 50.1 days (±11.4) (Table 3).

### Complications & death on ICU following initial ROSC

In one patient primary attempt for cannulation failed on the right/left side and was successfully on the contralateral lower limb.

Two patients out of those with ROSC had a local bleeding at the puncture site. After decannulation revision surgery and suture of the vessel was performed by a vascular surgeon. One patient suffered from a malpositioning of the Y-cannula causing ischemia of the affected leg and requiring fasciotomy after reperfusion.

One patient ECLS was decannulated after 11 days. After initial improving of her general state she died due to MOF and sepsis 45 days following trauma on ICU. The second patient was decannulated in consent with the patient’s family due to poor prognosis after two days and died two days later due to MOF. The third patient died 2 days after ROSC due to intravascular diffuse coagulopathy and MOF (ISS 50). Treatment with ECLS was still ongoing.

The fourth patient was cardiovascular stable, however she sustained a hypoxic cerebral edema and died on the first day on ICU.

### Neurological outcome

The median GCS at admission was 3 (IQR 9.25, range 3–15). Mean GOS of the survivors was 5 (IQR 0.25, range 4–5) and the mean CPC was 1 (IQR 0.25, range 1–2) at discharge from hospital respectively. One of the survivors is hemiplegic and another one suffers from sensibility disorder at his right upper and lower limb.

## Discussion

ECLS remains the last option in patients when other treatment strategies fail^4,5,14,15,17^. In the current study one third of the patients survived with good neurological outcome. We found a significant correlation between age and survival in favor of patients of young age. Further, we observed a trend towards better survival in patients with a lower ISS and earlier cannulation.

Due to the literature an overall survival rate of 25–80% after traumatic cardiorespiratory and following ECLS treatment is reported^5,14,15,18^. The differences of the overall survival rates, might be due different observation periods and adapted treatment protocols within the last years^2,5,14,15^. Some authors reported in comparable studies a promising initial successful treatment with ECLS in 70–80% of the patients, however the number of survivors decreased to 27% within the follow up^5,15^. Grubmuller *et al*.^17^ reported that a higher ISS in patients with chest trauma is not associated with inferior survival due to modern diagnosis and treatment options. In contrast, our data show that a higher ISS is indeed associated with an increased mortality rate. This finding in in accordance to the data reported by Bonacchi *et al*. who found that patients with a higher ISS had a higher failure rate of ECLS.

Currently, there are several recommendations for time to ECLS cannualtion in ARDS or acute cardiopulmonary failure^12,14,15,19,20^. Huh *et al*. reported an average time of 142 ± 48.2 minutes from initial trauma to veno-arterial ECLS in their patients with traumatic cardiac arrest. They suggested that the high survival of 80% was supported from early cannulation to veno-arterial ECLS^5^. An European group reported an average time to ECLS from trauma of about 6 hours in patients with successful and failed cannulation for ECLS^15^. A literature review by Bonnachi *et al*.^14^ reveals that a delayed application of ECLS in patients with cardiac impairment or shock might contribute to further tissue hypoperfusion leading to irreversible intravascular diffuse coagulopathy and death. However, the authors strongly recommend heparin-free ECLS in severely injured patients due to impaired blood coagulation^14^. In contrast it was reported that early cannulation (<24 h) in patients with traumatic ARDS is associated with higher risk for major bleedings or other complications and was associated with a higher mortality^8,19^. In comparison to the prior reported data, the time to cannulation was in average earlier in the current study^5,14,15^. Survivors were cannulated earlier compared to non-survivors. This might indicate that earlier cannulation in traumatic cardiorespiratory failure is beneficial to improve survival.

The most common ECLS related complications were local bleeding and malposition of the Y cannula with accompanying ischemia of an extremity. In one patient (ISS 50) intravascular diffuse coagulopathy occurred within 48 hours. Cannulation for ELCS (heparin-free) was performed 3 hours after trauma and thoracotomy. These findings, are comparable to prior reported data^14,18^. Moreover, ECLS related complications did not effect the overall survival in the current study.

Data on neurological outcome of patients with ECLS for traumatic cardiopulmonary failure is rare^12^. Huh *et al*. reported good general outcome and two patients with cognitive or visual impairment after resuscitation and ECLS cannulation^5^. Kim *et al*. reported satisfying neurological outcome with an average CPC of 1 (1–2.5) in patients with ECLS following traumatic cardiorespiratory or traumatic ARDS^21^. In contrast Ryu *et al*. observed good neurological outcome in 59% and poor in 41% in patients following non-traumatic cardiopulmonary failure. High serum lactic acid levels and prolonged interval from cardiac arrest to ECLS had negative impact on neurological performance at discharge^22^. In the present study long-term survivors of traumatic cardiac arrest had a satisfying neurological performance at discharge from hospital, which might differ from current literature due to the small sample size^12,21^.

In summary, there are several limitations of this study. (1) We retrospectively assessed a small patient collective. (2) During the long observation period between 2002 and 2016 treatment protocols were adapted. (3) The heterogeneous patient population with different indications for ECLS (cardiac failure or hypoxemia) might have influence on the results. However, our results showed that ECLS is a valuable treatment in severely injured patients with traumatic cardiorespiratory failure and might improve survival with good neurological outcome. Younger patients had a significantly higher survival rate. Patients with a lower ISS were also associated with a higher survival rate. One third of the patients with ECLS survived in this study. Conversely, refrain form ECLS treatment would be the certain death of these patients since it is the last option. Consideration of earlier cannulation in traumatic cardiorespiratory failure might be beneficial to improve survival.

## Methods

This retrospective study analyzes all patients admitted with an ISS >16 to a Level I trauma center between 2002 and 2016. Inclusion criteria were ECLS in acute trauma treatment within ≤6 hours from injury due to cardiopulmonary failure with shock or acute respiratory insufficiency with severe hypoxemia. The cut off of <6 hours was defined as timespan between accident, rescue time until admission to hospital and advanced life support at the emergency trauma room. In total 853 trauma patients with an ISS >16 were admitted to our department. Eighteen patients (2.1%, 7 females, 11 male) with an average age of 31.6 ± 17.1 (range 1–64) required ECLS for acute cardiorespiratory failure.

Out of this collective 14 patients had a traumatic cardiac arrest, three patients had traumatic hypoxemia and one patient had traumatic cardiac failure and hypoxemia. Veno-arterial cannulation was performed in 15 patients (83.3%) with traumatic cardiac failure. Veno-venous cannulation was necessary in only 3 patients (16.7%) with acute hypoxemia. Nine patients (50%) underwent thoracotomy with cross-clamping of the aorta and open cardiac massage before ECLS cannulation. Detailed information on trauma mechanism, injuries and patient demographics are given in Table 1.

**Table 1:** Clinical Presentation & intial BGA (Blood Gas Analsysis).

	PH	pCO_2_ (mmHg)	pO_2_ (mmHg)	Lactate (mmol/L)	SaO_2_ (%)^#^	HF	RR	GCS
1	7,07	83,10	70,70	11,90	73	ALS	ALS	3
2	7,15	51,10	114,00	5,20	90	118	85/40	3
3	7,11	71,20	74.1	3,10	90	120	86/40	3
4	7,09	85,30	82,70	13,90	84	132	82/38	3
5	7,08	56,40	80,50	3,30	90	101	61/38	3
6	7,17	12,00	606,00	14,70	88	135	55/28	3
7	7,10	116,00	79,40	4,50	95	98	135/100	13
8	7,10	90,00	47,00	7,00	90	89	60/35	3
9	7,11	51,10	65,60	6,30	80	105	95/60	3
10	6,97	54,80	207,00	8,70	65	124	46/60	3
11	7,16	49,00	88,00	2,30	91	121	80/50	12
12	7,13	45,90	657,00	4,40	88	132	66/32	3
13	7,19	34,90	127,00	13,10	95	133	95/60	15
14	7,08	32,00	302,00	11,40	88	125	105/75	3
15*	7,18	61,00	39,10	2,30	80	77	108/52	15
16	7,23	119,00	91,20	1,20	70	110	115/75	13
17	7,15	68,90	68,20	2,40	90	100	100/80	15
18	6,96	124,00	99,40	1,00	80	135	115/75	3

HF: heart frequency, RR: blood pressure, GCS: Glasgow Coma Scale.

*Venous BGA, ^#^peripheral O_2_ saturation (pulse oximeter).

**Table 2:** Patient’s Demographics.

	Survivors	Non-survivors	p-value
Patients (n)	6	12	
**Sex**			
Female	2	5	n.s.
Male	4	7	n.s.
Age	19 IQR 23.5 (1–37)	32.5 IQR 30 (18–64)	0.0289
ISS	23.5 IQR 22.75 (16–50)	38.5 IQR 16.5 (12–50)	n.s.
SOFA	12 IQR 1.75 (9–13)	10 IQR 5 (5–15)	n.s.
GCS	7.5 IQR 12 (3–15)	3 IQR 2.25 (3–15)	n.s.
VA ECLS	4	11	
VV ECLS	2	1	
Time to ECLS (hours)	2 IQR 0,25 (1–2)	2 IQR 4 (1–6)	n.s.
Time of ECLS (hours)	48 IQR 54 (24–96)	0.5 IQR 27 (0–264)	n.s.
Patients (n)	6	12	n.s.
**Mortality**			
<24 hours		8	
24 hours		4	
GOS	5 IQR 0.25	1 ± IQR 0	0.0001
CPC	1IQR 0.25	5 IQR 0	0.0001
hospital stay	39.5 IQR 46.75 (28–98)	0 IQR 1.75 (0–45)	0.0002
ICU stay	25 IQR 34.75 (15–86)	0 IQR 1.75 (0–45)	0.0081
Complications	1 (16.7%)	2 (16.7%)	n.s.

*Time to; MVA: motor vehicle accident; CPR: cardiopulmonary resuscitation, ECLS: Extracorporeal Membrane Life Support VA: Veno-arterial, VV: Veno-venous.

**Table 3:** Survivors vs Non-Survivors.

Patient	Age	Sex	ISS	Admis-sion^*^	ECLS^*^	ECLS	Trauma mechanism	Resuscitation	Cardiac failure	Hypox-emia	Cerebral injury	Abdomen Injury	Chest trauma	Pelvic injury	limb injury	ROSC	Sur-vival
1	23	f	32	15′	40′	va	MVA	thoracotomy	yes	—	—	—	yes	yes	yes	—	no
2	48	f	33	30′	55′	va	Fall >3 m	thoracotomy	yes	—	—	—	yes	yes	yes	—	no
3	18	m	44	25′	55′	va	Fall >3 m	CPR/ALS	yes	—	yes	yes	yes	yes	yes	—	no
4	41	f	16	25′	50′	va	gunshot	CPR/ALS	yes	—	—	yes	—	—	—	—	no
5	61	f	41	40′	95′	va	Fall >3 m	CPR/ALS	yes	—	yes	yes	yes	—	yes	—	no
6	55	m	27	25′	70′	va	MVA	CPR/ALS	yes	—	—	yes	—	yes	yes	—	no
7	64	m	18	45′	110′	va	MVA	CPR/ALS	—	yes	—	—	yes	—	yes	yes	no
8	30	m	41	45′	115′	vv	Fall >3 m	CPR/ALS	yes	yes	yes	yes	yes	yes	yes	—	no
9	29	m	50	50′	170′	va	MVA	thoracotomy	yes	—	—	.	yes	yes	yes	yes	no
10	35	m	48	45′	185′	va	MVA	thoracotomy	yes	—	yes	—	yes	—	yes	yes	no
11	24	f	45	75′	330′	va	MVA	thoracotomy	yes	—	—	yes	yes	—	—	yes	no
12	23	m	36	55′	345′	va	Stabbing	thoracotomy	yes	—	—	—	yes	—	—	—	no
13	20	m	16	25′	60′	va	Stabbing	thoracotomy	yes	—	—	yes	yes	—	—	yes	yes
14	30	m	17	30′	110′	va	Contusion	thoracotomy	yes	—	—	yes	yes	—	—	yes	yes
15	18	m	36	40′	130′	va	MVA	thoracotomy	yes	—	—	—	yes	yes	yes	yes	yes
16	11	f	18	35′	115′	vv	MVA	CPR/ALS	—	yes	—	—	yes	—	yes	yes	yes
17	37	m	50	35′	125′	va	MVA	CPR/ALS	yes	—	—	—	yes	—	yes	yes	yes
18	1	f	29	34′	120′	vv	MVA	CPR/ALS	—	yes	yes	—	yes	—	—	yes	yes
total									15	4	5	7	16	7	12	10	6

ECLS: Extracorporeal Membrane Life Support, VA: Veno-arterial, VV: Veno-venous, SOFA: Sequential Organ Failure Assessment-Score, GCS: Glasgow Coma Scale, GOS: Glasgow Outcome Scale, CPC: Cerebral Performance Category Score).

### Diagnosis and treatment

For acute emergency trauma room management, a standardized clinical body check, FAST ultrasound scan, computer tomography and extremity radiographs were used. Advanced life support, including vasoactive medications and endotracheal intubation, was performed in accordance with the European Resuscitation Council Guidelines^23^. Blood gas analysis (BGA) and basic blood samples including clothing parameters, red blood count, renal and liver function were taken within the stabilization phase in the trauma room. Shock was defined as lack of sufficient tissue oxygenation due to decreased circulating blood volume, cardiac function, or systemic vascular resistance.

Followed by drop in blood pressure to less than 60 mmHg resulting that vital organs cannot be perfused sufficiently 24. Hypoxemia was defined as peripheral oxygen saturation <85% or blood gas analysis showed a PaCO_2_ > 115 mmHg/PaO_2_ < 50 mmHg.

ECLS cannulation was indicated in the presence of following conditions: thoracic trauma with impaired oxygenation and hypoxemia, imminent or actual traumatic cardiac arrest under ongoing ALS with or without thoracotomy and open cardiac massage without signs of ROSC or improvement of oxygenation. In the presence of one of these conditions, an early cannulation to ECLS was aimed.

The final decision for cannulation was made by the trauma leader. The femoral vessels were used for the veno-arterial cannulation. The cannulation was performed using the percutaneous Seldinger technique with ultrasound guidance or by a small incision to cannulate directly the vessel. In addition, the cannulation was secured by a vascular suture. For the veno-venous-ECLS a femoro-jugular cannulation was performed using an ultrasound guided percutaneous Sedlinger’s technique. Blood clotting was checked in all patients before beginning of ECLS and heparin was used as anticoagulation. Infants requiring veno-venous ECLS, cannulation was performed femoro-jugular.

Depending on blood coagulation and severity of injuries, heparin-free ECLS, delaying heparin administration for up to 48 hours, or an initial bolus of 500IE/h heparin was started soon after transfer to ICU. A therapeutic level of the aPTT of 60–65 seconds was aimed. LDH was checked as a surrogate parameter for hemolysis to reduce heparin dose if necessary.

### Outcome parameters

The analysis of ECLS included indication for ECLS, type of ECLS, time to ECLS, duration of ECLS, length of ICU and hospital stay. All ECLS treatment and trauma related complications including bleeding, thrombosis, line infection, vascular injury, cannula dislocation, pneumonia, sepsis or further surgical intervention were recorded. The Injury Severity Score (ISS) and the Glasgow Coma Scale (GCS) were routinely assessed to determine neurological status and impact of trauma at time of admission. The Sequential Organ Failure Assessment-Score (SOFA-Score) was assessed initially after the first blood samples were analyzed to predict likelihood of mortality. For neurological outcome the Glasgow Outcome Scale (GOS) and the Cerebral Performance Category Score (CPC) were done at time of hospital discharge.

### Complications

All ECLS, further ALS and trauma related complications such as bleeding, thrombosis, line infection, vascular injuries and cannula dislocation were recorded.

### Statistical analysis

Due to small sample size and non normally distributed variables we used the Mann-Whitney-U-Test statistical analysis of quantitative data. Statistical significance was set at α = 0.05. All calculations were performed with Microsoft Excel® (Microsoft Corporation, Redmond, WA, USA) and SPSS® (Version 24.0, SPSS Inc., Chicago, IL, USA).

### Ethical approval

This article does not contain any interventional studies on human or animal participants performed by any of the authors (Ethics Committee of the Medical University of Vienna 1560/2016). All experimental protocols were approved by Ethics Committee of the Medical University of Vienna. Informed consent was not required by Ethics Committee of the Medical University of Vienna.
